# Beyond the binary description of sinus tracts in periprosthetic joint infection

**DOI:** 10.5194/jbji-11-651-2026

**Published:** 2026-10-16

**Authors:** Irene K. Sigmund, Marjan Wouthuyzen-Bakker, Tristan Ferry, Ricardo Sousa, Daniel Pérez-Prieto, Alex Soriano, Volker Alt, Willem J. Metsemakers, Martin Clauss, Rihard Trebse

**Affiliations:** 1 Department of Orthopaedics and Traumatology, Medical University of Vienna, Vienna, Austria; 2 Department of Medical Microbiology and Infection Prevention, University Medical Center Groningen, University of Groningen, Groningen, the Netherlands; 3 Infectious and Tropical Diseases Unit, Croix-Rousse Hospital, Hospices Civils de Lyon, Université Claude Bernard Lyon 1, Lyon, France; 4 Porto Bone Infection Group (GRIP), Orthopaedic Department, ULS Santo António, Porto, Portugal; 5 Hospital del Mar, Barcelona, Spain, Autonomous University of Barcelona, Barcelona, Spain; 6 Hospital Clinic of Barcelona, Barcelona, Spain; 7 IDIBAPS, Barcelona, Spain; 8 CIBERNIF, Ciber in Infectious Diseases, ISCIII, Madrid, Spain; 9 Department for Trauma Surgery and Regensburg University Center for Muskuloskeletal Infections, University Hospital Regensburg, Regensburg, Germany; 10 Department of Trauma Surgery, University Hospitals Leuven, Leuven, Belgium; 11 Department of Development and Regeneration, KU Leuven, Leuven, Belgium; 12 Center for Musculoskeletal Infections (ZMSI), Department of Orthopaedics and Trauma Surgery, University Hospital Basel, Basel, Switzerland; 13 Orthopaedic Hospital Valdoltra, Ankaran, Slovenia

## Abstract

Current periprosthetic joint infection (PJI) definitions classify sinus tracts as present or absent. While this binary approach is appropriate for diagnosing PJI, it does not capture the heterogeneity of sinus tracts relevant to surgical decision-making. We propose that future studies explore a standardized characterization of sinus tract complexity to better inform surgical treatment.

## 1 Viewpoint

The presence of a sinus tract communicating with the prosthesis is one of the most specific clinical signs of periprosthetic joint infection (PJI) (Luger et al., 2026) and is universally accepted as a confirmatory criterion in all major diagnostic definitions, including those of the Infectious Diseases Society of America (IDSA), the Musculoskeletal Infection Society (MSIS), the International Consensus Meeting (ICM), and the European Bone and Joint Infection Society (EBJIS) (McNally et al., 2021; Osmon et al., 2013; Shohat et al., 2019; Parvizi and Gehrke, 2014). Beyond its diagnostic importance, the presence of a sinus tract also influences surgical decision-making, as it is frequently considered a contraindication to debridement, antibiotics, and implant retention (DAIR) and, in many centres, may also affect the selection of patients for one-stage revision (Argenson et al., 2019; Bialecki et al., 2019; Osmon et al., 2013). Consequently, how a sinus tract is defined extends far beyond terminology, with implications for diagnosis, treatment selection, patient counselling, study design, and the interpretation of clinical outcomes. While this binary approach provides the specificity and reproducibility required for diagnosing PJI, it provides little information on the heterogeneity of sinus tracts or their potential implications for surgical decision-making.

Several classification systems have been proposed to describe the severity and complexity of PJI, including the McPherson classification (McPherson et al., 1999, 2002), JS-BACH (Hotchen et al., 2021), and the PJI-TNM classification (Alt et al., 2020). Both the JS-BACH and PJI-TNM classifications incorporate soft-tissue involvement as an important component of disease severity, yet neither specifically characterizes sinus tracts. In contrast, the McPherson classification recognizes a synovial-cutaneous fistula as one of the compromising factors of the local extremity grade. However, it does not further characterize different sinus tract phenotypes or provide standardized descriptors of sinus tract complexity that may be relevant for surgical planning and future research.

This lack of a standardized characterization is also reflected in current treatment recommendations. The 2013 IDSA guidelines recommend against DAIR or one-stage revision in patients presenting with a sinus tract and instead favour two-stage exchange (Osmon et al., 2013). In contrast, the 2018 International Consensus Meeting (ICM) stated that a complex sinus tract that cannot be excised together with the previous surgical scar should be regarded as a relative contraindication to one-stage exchange (Bialecki et al., 2019), without further defining what constitutes a *complex* sinus tract. More recently, the 2025 ICM concluded that one-stage revision may be considered in patients with a sinus tract provided that adequate soft-tissue coverage can be achieved at the conclusion of surgery (Hansen et al., 2025). Notably, this recommendation achieved only moderate consensus (70.7 % agreement, 24.7 % disagreement), highlighting the ongoing uncertainty regarding the surgical implications of sinus tract characteristics. Collectively, these recommendations implicitly acknowledge that not all sinus tracts are clinically equivalent, yet no consensus currently exists on how sinus tract complexity should be defined or reported.

Consequently, in clinical practice, surgeons routinely assess far more than the presence or absence of a sinus tract when planning treatment. The extent of soft-tissue involvement, the condition of the surrounding tissues, implant exposure, previous surgical procedures, and the overall reconstructive complexity all influence whether limb reconstruction is feasible and which surgical strategy is most appropriate. Two patients fulfilling the diagnostic definition of a sinus tract may therefore represent fundamentally different reconstructive problems. For example, a patient presenting with a single, small sinus tract, minimal soft-tissue involvement, and healthy surrounding skin differs substantially from a patient with multiple communicating fistulae, chronic drainage, extensive soft-tissue destruction, implant exposure, and previous surgical procedures. Although both fulfil the diagnostic definition of a sinus tract, they are unlikely to benefit from identical surgical strategies.

Rather than merely documenting the presence of a sinus tract, future clinical studies should systematically report its characteristics. The systematic reporting of these descriptors may help to explain the conflicting outcomes reported after one- and two-stage revision in patients presenting with a sinus tract (Kheir et al., 2018; Xu et al., 2024, 2019; Luger et al., 2026; Lausmann et al., 2025). While some studies have demonstrated encouraging eradication rates, others have reported substantially higher rates of treatment failure and reinfection. A binary description may therefore be insufficient to capture the complexity of disease relevant to surgical decision-making.

To facilitate standardized reporting, Table 1 proposes a set of standardized descriptors of sinus tract complexity intended as a hypothesis-generating minimum reporting dataset for future clinical studies. (These descriptors are not presented as a validated classification or treatment algorithm.)

**Table 1:**
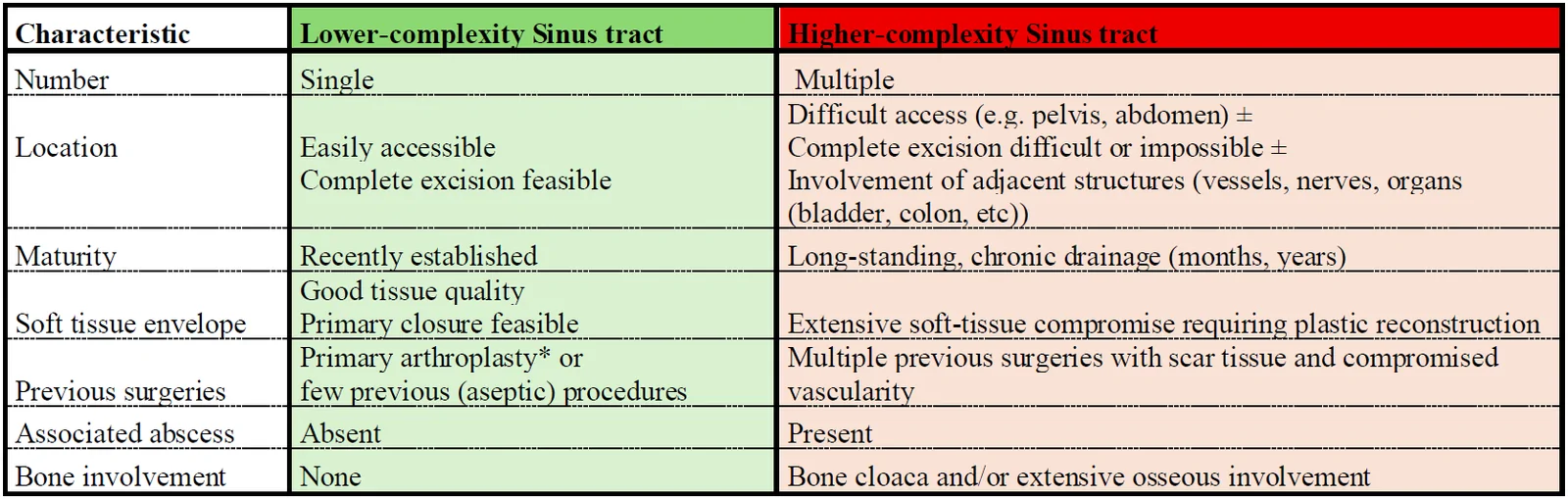
Proposed standardized descriptors of sinus tract complexity with illustrative examples of lower- and higher-complexity presentations. The proposed descriptors are intended to facilitate standardized reporting of sinus tract complexity, while the illustrative examples demonstrate its clinical spectrum. Neither should be interpreted as a validated classification or treatment algorithm.

^*^ Without multiple previous surgeries before the primary arthroplasty.

Characteristics of the sinus tract and the surrounding local disease – including number, location, maturity, soft-tissue compromise, implant exposure, previous surgical procedures, associated abscess formation, and osseous involvement (e.g. cloaca formation) – may collectively provide a more comprehensive characterization of sinus tract complexity (Table 1). These characteristics should be interpreted in the context of joint-specific anatomy and, where relevant, imaging and intraoperative findings, as the external appearance may underestimate the extent of underlying disease. In the absence of validated thresholds for duration or maturity, future studies should report these characteristics descriptively rather than applying arbitrary cut-offs. Importantly, these descriptors should not be regarded as additional diagnostic criteria or as an independent treatment algorithm. Rather, they represent a proposed set of standardized descriptors intended to facilitate the consistent reporting of sinus tract complexity in future clinical studies. Their individual prognostic significance remains to be established and should be evaluated in prospective validation studies.

It is equally important to recognize that sinus tract complexity represents only one component of the multifactorial complexity of PJI. Surgical decision-making should never be based on sinus tract characteristics alone but should always be interpreted in conjunction with other well-established factors, including the type and chronicity of infection, host status, microbiology, vascularity, bone involvement, implant characteristics, soft-tissue condition, and overall reconstructive feasibility. Future classification or staging systems may also consider incorporating standardized descriptors of sinus tract complexity in addition to existing assessments of soft-tissue involvement, including the feasibility of soft-tissue coverage or the need for reconstructive procedures. The aim is therefore not to replace existing classification systems but rather to complement them by incorporating standardized descriptors of sinus tract complexity into the overall assessment of PJI.

Building upon this concept, we propose that future clinical studies move beyond the current binary description of sinus tracts by systematically reporting standardized descriptors of sinus tract complexity. Such reporting may help to explain the heterogeneity in outcomes by allowing future studies to investigate how different sinus tract phenotypes interact with the selected treatment strategy. It may also enable meaningful comparisons between studies, improve risk stratification, and provide a common language for describing sinus tract complexity across centres. Future validation studies may refine the descriptors proposed here and determine their clinical relevance. If validated, they may evolve into a clinically useful framework that complements existing assessments of host, infection, bone, soft-tissue, and implant-related factors when planning treatment and may ultimately support the development of evidence-based staging systems that integrate sinus tract characteristics with these established domains.

Diagnosis begins with identifying a sinus tract; treatment requires understanding its complexity (in conjunction with the many other factors that contribute to the overall complexity of PJI).

## Data Availability

No data sets were used in this article.
